# Sport-specific balance tests account for youth alpine skiers’ ranking

**DOI:** 10.3389/fphys.2023.1205347

**Published:** 2023-07-20

**Authors:** Alex Rizzato, Nina Verdel, Antonio Paoli, Matej Supej, Giuseppe Marcolin

**Affiliations:** ^1^ Department of Biomedical Sciences, University of Padova, Padua, Italy; ^2^ Faculty of Sports, University of Ljubljana, Ljubljana, Slovenia

**Keywords:** postural control, alpine skiing, center of pressure, sport specificity, assessment

## Abstract

**Objective:** Alpine skiing requires complex motor skills and fine adjustments to maintain balance in dynamic and challenging conditions. This study aimed to understand whether the balance ability in unspecific (UST) and sport-specific (SST) tasks could depend on the skiers’ ranking level. The balance performance of the dominant and non-dominant limbs in the SST was also investigated.

**Methods:** Twenty-five skiers (14.96 ± 1.61 yrs; 1.69 ± 0.69 m; 59.9 ± 9.52 kg) were divided into high-ranking (position < 50) and low-ranking (position > 50) groups. Subjects performed three balance conditions: static (ST), dynamic UST, and dynamic SST. Subjects stood on an unstable board over a force platform during UST. During SST, subjects wore ski boots, grasped ski poles, and each foot was clipped to an unstable board over two force plates. From the center-of-pressure (CoP) trajectory the area of the 95th percentile ellipse and the CoP mean velocity were calculated. Angular displacements were recorded by a 12-camera system, to calculate the full balance (FB), fine (FiB), and gross (GB) balance in UST and SST.

**Results:** Balance control was higher (*p* < 0.01) in high-ranking than low-ranking skiers only in the SST. Kinematic parameters (i.e., FB, FiB, and GB) showed a higher (*p* < 0.001) balance performance in SST than UST independently from the group. Dominant and non-dominant limbs motion was similar (Pearson correlation, r = 0.97) in SST independently from the skiers’ ranking.

**Conclusion:** High-ranking skiers showed better balance control and performance than low-ranking skiers only when the task was sport-specific. Therefore, we suggest testing balance under sport-specific conditions to discriminate the youth skiers’ abilities.

## Introduction

Balance is considered a multifactorial motor skill, including input from sensory systems (i.e., proprioceptive, vestibular, and visual) and output responses from different parts of the nervous system and muscles (Horak et al., 1997). Balance regulation involves reflex, automatic and cognitive processes, with a degree of progressive awareness in the response to goal-oriented tasks (Paillard, 2017; Rizzato et al., 2021). The center of pressure (CoP) displacement, derived from force platforms, represents the most reliable static balance assessment measure. Effective balance performance is a good prerequisite for improving the control of voluntary movements in sports and, consequently, enhancing athletic performance (Andreeva et al., 2021).

However, balance performance is influenced by the specific motor skills and environment of a sport that determine the athlete’s postural adaptation and strategies (Paillard, 2014). In balance regulation, training practice could differentiate the ability to use proprioceptive and visual-vestibular information (Golomer et al., 1999; Perrin et al., 2002) and could influence the strategies for modulating short or long neuronal loops (Paillard, 2014). Furthermore, results from prospective studies demonstrated that balance training can be a useful adjunct to the usual training of non-elite athletes to enhance motor skills (Kean et al., 2006; Yaggie and Campbell, 2006).

Balance performance is also affected by the physical exertion and sport-specific postures assumed during training and competitions. In this regard, a greater postural sway (i.e., poorer balance performance) was observed after an ultra-marathon running due to fatigue (Marcolin et al., 2016) or after activities requiring specific technical skills, such as gymnastics (Marcolin et al., 2019) and skiing (Zemková, 2014).

In some sports (e.g., soccer players, rifle shooters, and golfers), balance ability has been associated with competition level, with the more skilled athletes showing the best balance performance (Hrysomallis, 2011). Nevertheless, few studies analyzed subjects’ balance performance to distinguish the skill level among highly skilled athletes in a sport, and results are still inconsistent. For example, Era et al. (1996) showed better balance performance in highly trained male sport shooters at the international level than the national level. Conversely, Paillard et al. (2002) showed that balance performance was similar for judo athletes competing at a regional, national, and international level. Vuillerme et al. (2001) showed similar balance performance between gymnasts and non-gymnasts when visual cues were available. Marcolin et al. (2019) showed that the level of expertise had no effect on balance performance during a static task, whereas a sport-specific task was more selective for the level of expertise of young gymnasts. Thus, if balance is sport specific, it should influence the athletes’ skill level in sports discipline, even more so in disciplines where complex motor skills are performed. Nonetheless, the specificity of balance remains a widely-debated topic among researchers.

As with the sports above, alpine skiing involves specific physical demands in addition to skiing technique, including balance control (Neumayr et al., 2003; Maffiuletti et al., 2006; Hydren et al., 2013; Polat, 2016; Gilgien et al., 2018). Furthermore, in alpine skiing turning, there are large asymmetries in ground reaction forces between the inside and outside leg. However, these asymmetries are not related to asymmetries in skiers’ muscular strength (Ogrin et al., 2021). Alpine skiing requires also complex motor skills and fine adjustments to maintain balance in challenging conditions (Schaff and Hauser, 1989; Raschner et al., 2017). Nevertheless, no study has investigated a possible effect of balance asymmetries between the two limbs on skiing tecqnique. Even if external conditions do not change suddenly and unexpectedly, the technique involved is similar to open motor skill sports due to the extremely high variability of the skiing conditions (Gilgien et al., 2018). For example, course setting, terrain, snow conditions, speed, and visibility can all affect skiers’ performance (Supej and Holmberg, 2019).

In alpine skiing, wearing ski boots could affect balance control due to ankle joint constraints (Zemková, 2014). Laboratory studies found that experienced skiers can adjust their muscle coordination to maintain balance when wearing ski boots (Noé et al., 2009). In a similar setting, skiers showed better medio-lateral sway than young fit subjects standing on dynamometric platform with ski boots on (Zemková, 2014). However, off-snow balance training usually imitates skiing without wearing ski boots. Although the aforementioned studies examined the skiers performing simple and sport-specific tasks with and without ski boots (Noé et al., 2009; Zemková, 2014), the major limitation was that skiers’ feet were on a single base of support without ski bindings. This experimental arrangement did not correspond to real skiing, where each boot is bound to its own ski.

Therefore, the present study aimed to investigate whether the balance ability in unspecific and sport-specific tasks could depend on the skiers’ ranking. Assuming that balance is specific to a sport discipline, we expected differences in balance performance between high-ranking and low-ranking skiers to occur only when the balance task is sport-specific. Considering that each boot was clipped to an independent base of support in the sport-specific task, we expected a better balance performance from the dominant than the non-dominant limb.

## Material and methods

### Subjects

Twenty-five young elite skiers volunteered to participate in the study (Table 1). They were divided into two groups based on the International Ski Federation ranking: high-ranking (i.e., chart position < 50 p; n = 13) and low-ranking (i.e., chart position > 50; n = 12). All the skiers were involved in national competitions at the time of the recruitment. The researchers screened subjects eligible for the study through an interview. Thus, skiers with no history of 1) orthopedic injuries in the last year, 2) neurological diseases, and 3) sight, hearing, or vestibular non-corrected disorders were enrolled in the study.

**TABLE 1:** Skiers’ characteristics. F = females; yrs = years.

	Subjects	Age (yrs)	Mass (kg)	Height (m)	Practice (yrs)
High-ranking	13 (F = 5)	15.46 ± 1.45	66.39 ± 7.12	1.72 ± 0.55	7.86 ± 1.81
Low-ranking	12 (F = 5)	14.41 ± 1.67	53.04 ± 6.44	1.65 ± 0.68	7.58 ± 2.77

The experimental protocol was approved by the Human Ethical Committee of the Department of Biomedical Science of the University of Padova and adhered to the principles of the Declaration of Helsinki; all precautions were taken to protect the skiers’ privacy. All the subjects involved in the study (i.e., skiers, parents, and coaches) were informed about the methods and aims of the study, gave their written informed consent, and had the option to discontinue the study at any time.

### Experimental design and measurement equipment

**FIGURE 1:**
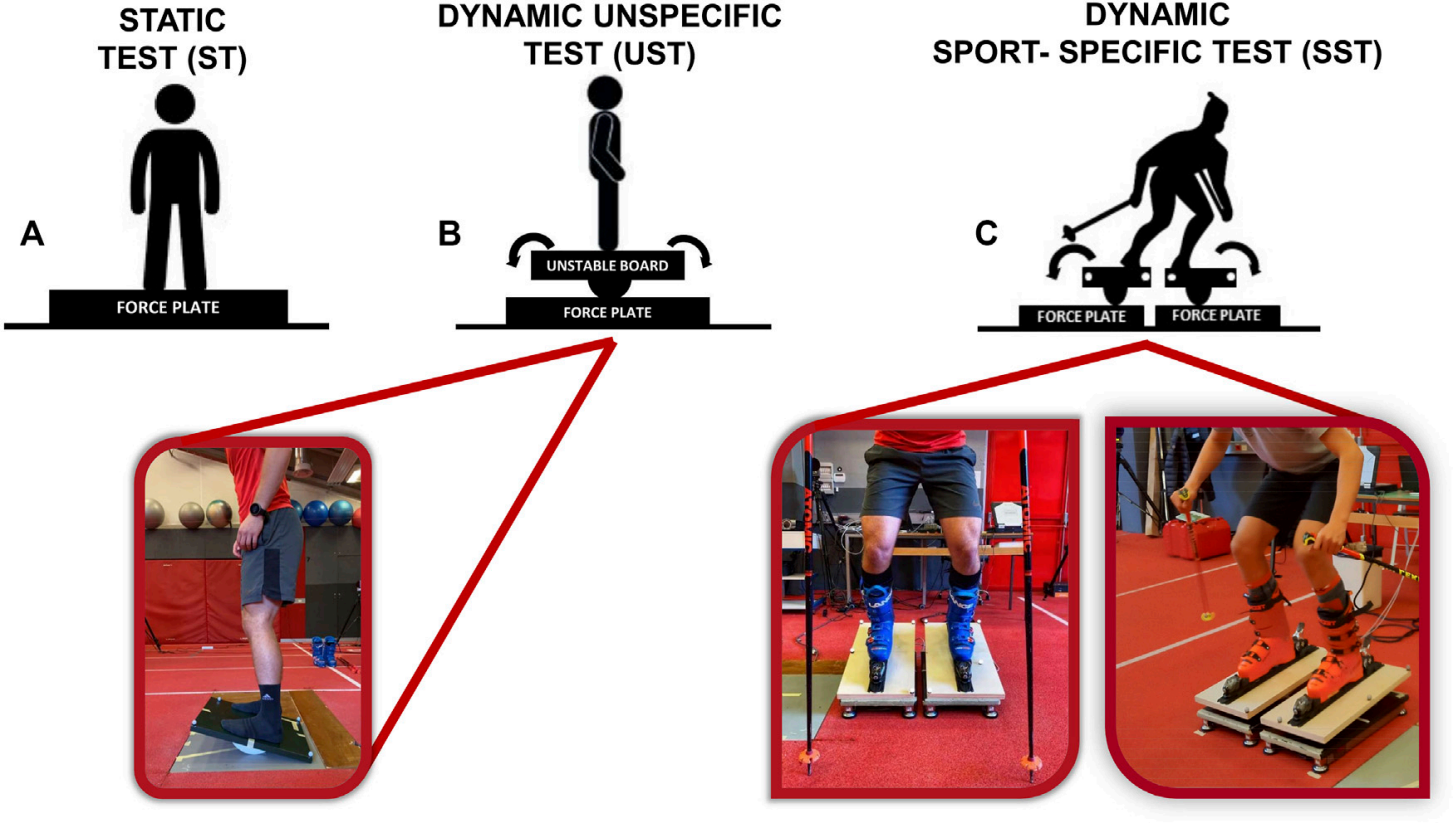
Experimental design. Static balance test **(A)**; Dynamic unspecific test **(B)**; Dynamic sport-specific test **(C)**.

An outlined cross-sectional design was employed (Figure 1) in which postural balance control was tested with eyes open under three conditions: static (ST), dynamic unspecific (UST), and dynamic sport-specific (SST). Static postural balance (Figure 1A) was assessed on a force platform (model: BP600600, AMTI, Watertown, MA, United States), requiring subjects to maintain the same static upright posture. The sampling frequency was set to 300 Hz to match dynamic conditions. Subjects were instructed to stand with legs extended and arms held at their sides in a natural posture. The position of feet on the force platform was standardized using a V-shaped frame, maintaining a distance of 7 cm between the heels and an angle of 30° between the two feet. This position was in accordance with the recommendations of the International Society of Posturography (Kapteyn et al., 1983).

During the UST condition (Figure 1B), subjects had to stand with parallel feet on an unstable square board (length: 50 cm; width: 50 cm; height: 8.5 cm; radius: 10.95 cm), which rotated along a single axis. A marine plywood semicylinder allowed the board to rotate 16° anteriorly and posteriorly. The unstable board was positioned over the AMTI force platform to collect the CoP trajectory at a 300 Hz sampling rate. Subjects were asked to maintain the board parallel to the ground as much as possible without moving the feet from their original position. During the test, subjects gazed at a line target vertically placed in front of them at approximately 80 cm keeping their hands on hips to standardize counterbalance actions.

During the dynamic sport-specific condition (Figure 1C), subjects wore their ski boots, held ski poles, and stood on two independent unstable boards (length: 60 cm; width: 30 cm; height: 8.5 cm; radius: 20.55 cm). Ski boots were clipped to ski bindings integrated into the unstable boards. A semicylinder made of marine plywood allowed the boards to rotate 16° in the anterior-posterior direction. The unstable boards were positioned over a bilateral force platform (S2P, Science to Practice Ltd., Ljubljana, Slovenia) that recorded data at 300 Hz. Before the test, subjects were required to touch the ground with the ski poles to find balance. Once the test began, they had to lift the ski poles from the floor and were allowed to move them for balance without touching the ground.

Under both dynamic conditions (i.e., UST and SST), four reflective markers were attached at the vertices of the unstable boards, and a 12-camera optoelectronic system (model: Oqus 7+: Qualisys, Gothenburg, Sweden) recorded their three-dimensional trajectories synchronously with the force platform at 150 Hz.

The signals from the Qualisys cameras as well as the AMTI force plate were recorded in Qualisys Track Manager (QTM, Qualisys, Gothenburg, Sweden); while the bilateral S2P force plate was connected to the Dewesoft Dewe 43 analogue-to-digital converter and recorded in the corresponding DewesoftX software (both Dewesoft, Trbovlje, Slovenia). All measuring devices were synchronized with a transistor-transistor logic (TTL) signal.

In total, subjects performed the static test before dynamic tests and three trials of 30 s for the other experimental conditions. Prior to the UST and SST conditions, skiers performed a 10 min-standardized warm-up (i.e., joint mobility and 10 repetitions of the half-squat exercise) and a 5-min familiarization to the unstable boards. Since dynamic tests were administered based on an increasing level of specificity, UST and SST were not randomized. The duration of the trials was set according to Scoppa and colleagues’ guidelines on stabilometric tests over force platforms (Scoppa et al., 2013). The rest between the trials was set to 60 s to allow for full recovery.

### Data analysis

**FIGURE 2:**
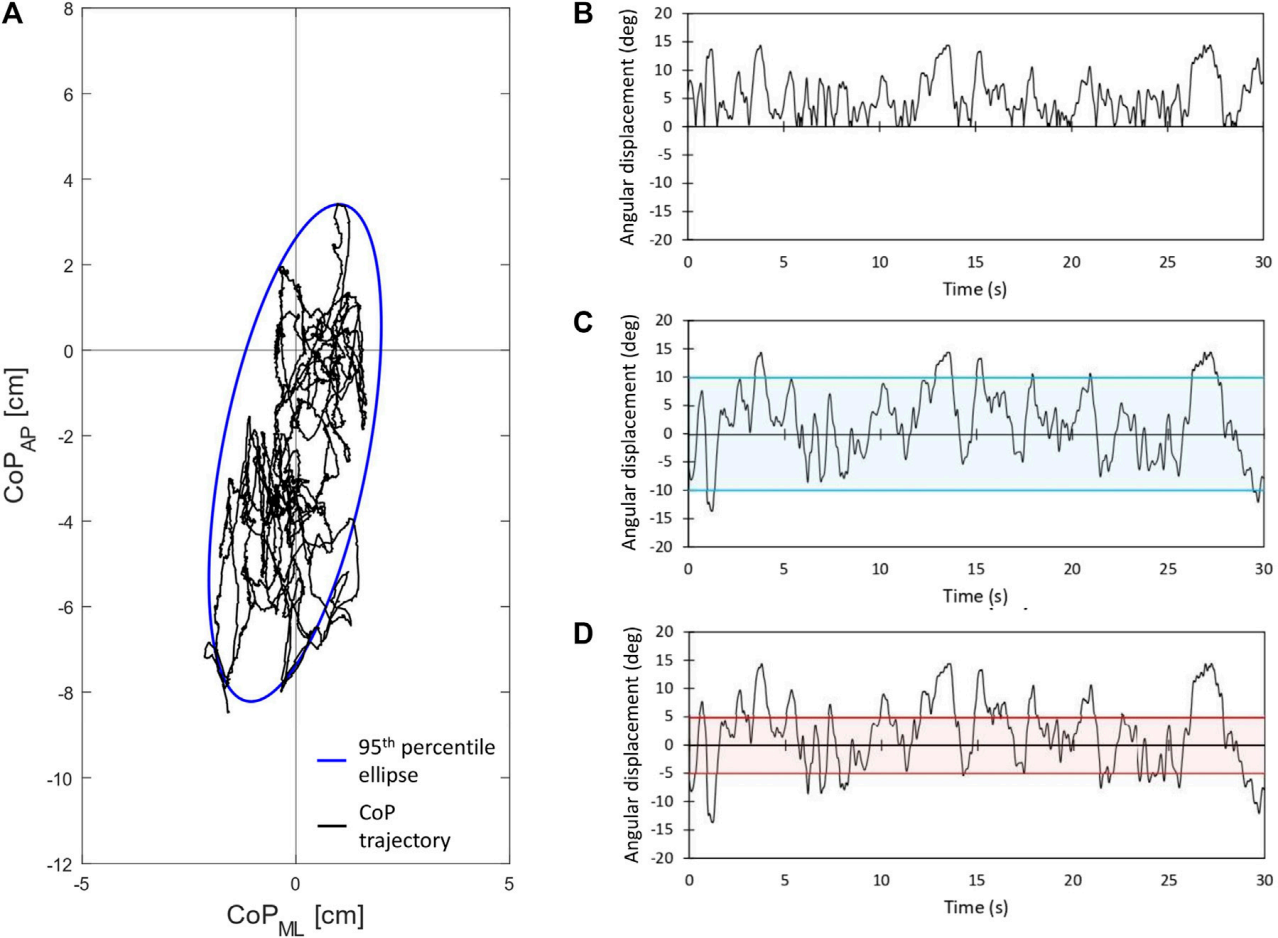
Graphical representation of the CoP-related and kinematics parameters. On the left: CoP trajectory and 95th percentile ellipse **(A)**. On the right: Full Balance **(B)**, Fine Balance **(C)**, and Gross Balance **(D)**.

The CoP trajectory was calculated from force data. Based on the CoP trajectory, two parameters were calculated: Area95 (the area of the 95th percentile ellipse measured in cm^2^) and Unit Path (the path length per time unit, i.e., the average CoP velocity, measured in cm∙s^−1^). A graphical representation of the CoP trajectory and of the 95th percentile ellipse is given in Figure 2A. In the SST, starting from the single-foot CoP trajectories (i.e., right and left feet), the whole-body CoP-related parameters were calculated with a customized script in the DewesoftX software. The four reflective markers placed on the lateral and medial edges of the unstable boards (the unique position of the four markers allowed the artificial intelligence in QTM to recognize each board) enabled the calculation of the angle of rotation of the square board: when the markers were parallel to the floor, the angle was 0°. Positive and negative angle values were measured when the unstable board rotated as a consequence of plantarflexion and dorsiflexion, respectively. As previously reported (Rizzato et al., 2023), three parameters were calculated to assess the dynamic balance performance: Full Balance (FB) (Figure 2B), Fine Balance (FiB) (Figure 2C), and Gross Balance (GB) (Figure D). FB is the integral of the angular-displacement curve and it is considered an index of the overall postural performance; FiB is time spent between +5° and −5°, as an index of fine-tuning balance adjustments; GB is the time spent between +10° deg and −10°, as an index of gross-tuning balance adjustments. The analysis tool was developed with MATLAB R2019a (The MathWorks, Inc., MA, United States).

### Statistical analysis

The *a-priori* power analysis calculation (G * Power 3.1.9.2 software) showed that a sample size of 24 participants and a medium effect size of 0.25 would have provided a statistical power of 0.7. The Shapiro-Wilk test was used to test the normality distribution of data. A two-way mixed-model analysis of variance (ANOVA) was used to investigate the significant main effect of the balance condition (i.e., ST, US, and SS) and the group (i.e., high-ranking *vs*. low-ranking skiers) or any interaction between them. The same statistical model was used to investigate the main effect of limb dominance (i.e., dominant *vs*. non-dominant) and group (i.e., high-ranking *vs*. low-ranking) on the Area95 and Unit Path parameters. In case of a statistically significant main effect or interaction, the Holm-Bonefrroni *post hoc* test was performed. Finally, Pearson correlation analysis was performed for each subject to study differences between the dominant and non-dominant limb, considering angular displacement strategies to face the SST condition. Subsequently, Pearson correlation coefficients (r) were reported as group mean ± SD. The strength of the correlation was interpreted as follows: weak (r ≤ 0.35), moderate (0.36 < r < 0.67), high (0.68 < r < 0.90), and very high (r ≥ 0.90) (Taylor, 1990). The significance level was set at *p* < 0.05. JASP Software, version 0.16.3.0, was used for statistical analysis.

## Results

**FIGURE 3:**
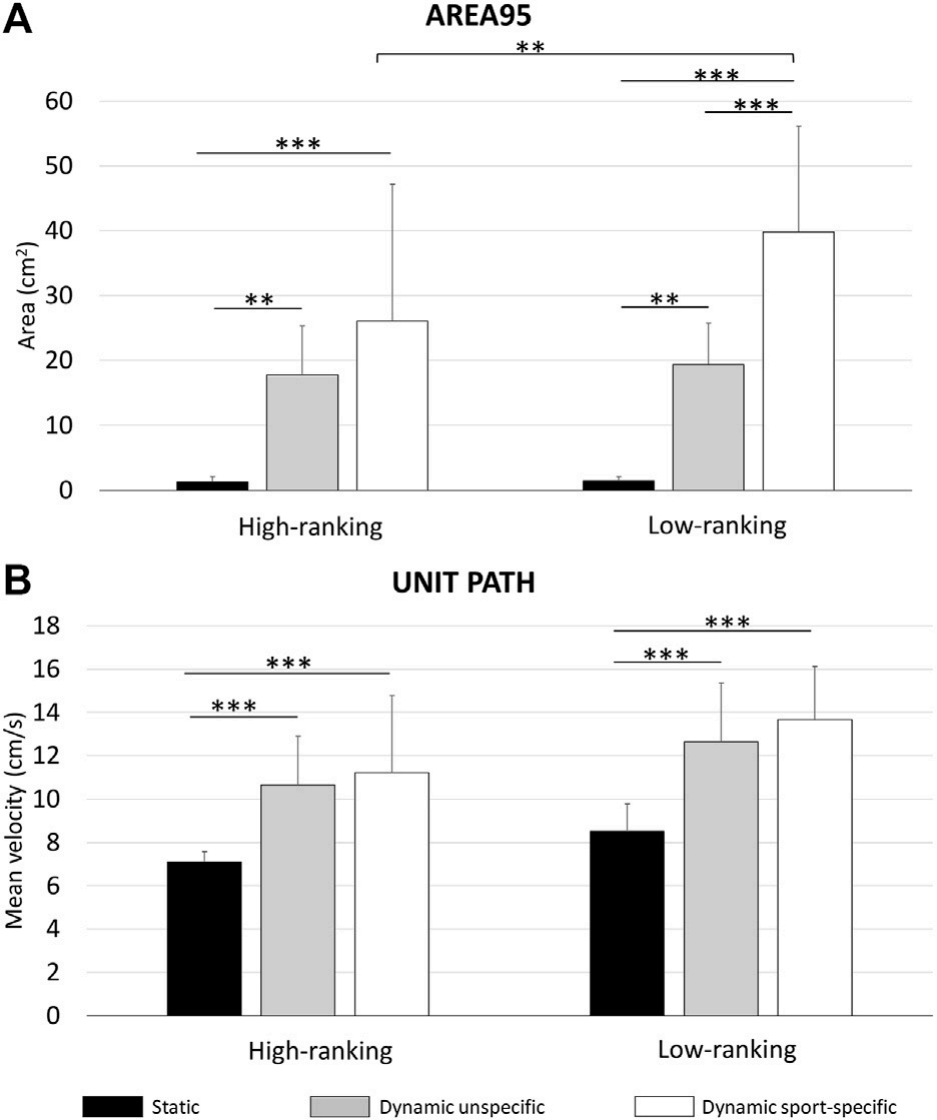
Area95 **(A)** and Unit Path **(B)** results in the ST, SST, and UST both for high-ranking and low-ranking skiers; data are presented as mean ± standard deviation. Significantly different ** (*p* < 0.01); *** (*p* < 0.001).

The results for the Area95 parameter in the ST, UST, and SST are presented in Figure 3A. The statistical analysis showed a significant main effect of the balance condition (*p* = 0.001; F = 56.82; ηp2 = 0.712) and an interaction balance *vs*. group (*p* = 0.05 F = 3.17; 0.05; ηp2 = 0.121). In detail, the Holm *post hoc* comparisons, displayed in Figure 3A, highlighted the differences among tests and between groups. The high-ranking skiers showed lower values than low-ranking skiers (between-group differences expressed in percentage for ST: −11.81%, UST: −8.43%, and SST: −34.83%).

The results for the Unit Path in the static and dynamic balance conditions are shown in Figure 3B. The statistical analysis showed a main effect of group (*p* = 0.008; F = 8.40; ηp2 = 0.268) and balance condition (*p* = 0.001; F = 37.12; ηp2 = 0.617). The Holm *post hoc* comparisons, displayed in Figure 3B, highlighted the differences among tests and between groups. The high-ranking skiers showed lower values of the Unit Path parameter than low-ranking skiers (between-group differences expressed in percentage for ST: −16.67%, UST: −15.60%, and SST: −17.94%).

The parameters FB, FiB, and GB and their statistical evaluation in the UST and SST dynamic balance test are summarized in Table 2. The statistical analysis showed a significant main effect of the balance condition (*p* = 0.001; F = 18.637; ηp2 = 0.448) and group condition (*p* = 0.05; F = 4.02; ηp2 = 0.149) for the FB. Similarly, a statistically significant main effect of the balance condition (*p* = 0.001; F = 12.60 ηp2 = 0.354) and group condition (*p* = 0.02; F = 5.63; ηp2 = 0.197) was detected for the GB. Only a significant main effect of the balance condition (*p* = 0.001; F = 13.44; ηp2 = 0.369) was observed for the FiB. Table 3 presented the effect sizes of the differences for all the balance parameters considered.

**TABLE 2:** Full Balance (FB), Fine Balance (FiB), and Gross Balance (GB) results in the SST and UST conditions for both high-ranking and low-ranking skiers. ***significantly different from UST (*p* < 0.001); # significantly different from high-ranking group (*p* < 0.05). Δ *% =* between-group differences expressed in percentage.

	Full balance (deg./s)		
	High-ranking	Low-ranking#	**Δ%**
Dynamic unspecific (UST)	154.48 ± 26.80	168.31 ± 29.40	−8.21
Dynamic sport-specific (SST)	114.72 ± 37.02***	137.02 ± 27.24***	−16.28

	Fine Balance (s)		
	High-ranking	Low-ranking	Δ%
Dynamic unspecific (UST)	17.59 ± 2.40	16.04 ± 2.57	+9.66
Dynamic sport-specific (SST)	21.13 ± 4.57***	19.86 ± 3.83***	+6.39

	Gross Balance (s)		
	High-ranking	Low-ranking#	**Δ%**
Dynamic unspecific (UST)	26.46 ± 1.97	25.08 ± 2.13	+5.50
Dynamic sport-specific (SST)	28.23 ± 1.68***	26.77 ± 1.93***	+5.45

**TABLE 3:** Effect size of the differences (Cohen’s d) for the Area95, Unit Path, Full Balance (FB), Gross Balance (GB), and Fine Balance (FiB). UST = unspecific test; SST = sport-specific test.

	Cohen’s d				
	Area95	Unit path	FB	GB	FiB
UST *vs*. SST	−1.232	−0.335	1.165	−0.896	−1.060
High-ranking *vs*. Low-ranking	−0.444	−0.826	−0.593	0.738	0.405

**FIGURE 4:**
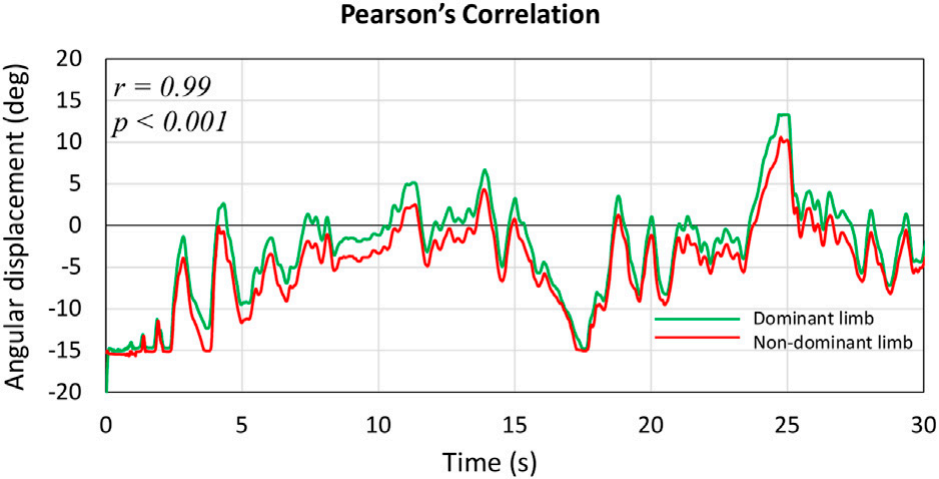
Pearson correlation between dominant (green line) and non-dominant (red line) limb angular displacement in the sport-specific test. Graph of one representative skier.

Results for balance control (i.e., Area95 and Unit Path) and balance performance (i.e., FB, FiB, and GB) of dominant and non-dominant limbs in the SST are presented in Table 4. The statistical analysis did not show significant main effects or interactions for the FB (*p* = 0.13), FiB (*p* = 0.56), and GB (*p* = 0.92) parameters. Finally, Pearson’s analysis showed for each subject a very high (*r* mean = 0.97 ± 0.018) and significant correlation (*p* < 0.001) between dominant and non-dominant limb board-related kinematics in the SST. Figure 4 shows the dominant and non-dominant angular displacement of one representative skier of our sample during the sport-specific task.

**TABLE 4:** Results of balance control (i.e., Area95 and Unit Path) and balance performance (i.e., Full balance, Fine Balance, and Gross Balance) between dominant and non-dominant limbs in the sport-specific test. Δ *% =* between-group differences expressed in percentage.

	High-ranking		Low-ranking		Δ%	
	Dominant	Non-dominant	Dominant	Non-dominant	Dominant	Non-dominant
Area95 (cm^2^)	20.30 ± 12.04	21.85 ± 15.69	31.11 ± 16.36	27.56 ± 12.95	−34.75	−20.71
Unit Path (cm/s)	10.59 ± 2.47	10.96 ± 3.32	13.12 ± 2.89	13.23 ± 2.68	−19.29	−17.15
FB (deg./s)	114.41 ± 38.40	111.83 ± 32.45	138.69 ± 31.99	127.46 ± 29.35	−17.51	−12.26
FiB (s)	15.38 ± 5.37	14.37 ± 3.62	13.82 ± 3.39	14.21 ± 3.87	+11.28	+1.12
GB (s)	24.42 ± 3.06	23.71 ± 4.14	23.96 ± 3.26	24.38 ± 2.65	+1.91	−2.74

## Discussion

This study aimed to evaluate skiers’ static and dynamic balance control to examine whether their ability to perform unspecific and sport-specific balance tasks could correspond to their ranking level. The main finding of our study supported the hypothesis that high-ranking skiers had a better balance ability than low-ranking skiers only when the task was sport-specific. Moreover, regardless of the skiers’ ranking, our findings did not confirm a better balance performance of the dominant than the non-dominant limb in coping with the sport-specific task.

The present results did not show any significant difference in static balance between high-ranking and low-ranking skiers. However, this finding is somewhat expected and not of high importance, since in Australian footballers, the static balance performance was poorly associated with the dynamic balance performance (Hrysomallis et al., 2006). Thus, we did not suggest inferring dynamic balance ability from static balance results, because previous findings on balance control did not support the transfer of such motor skills (Vuillerme et al., 2001; Asseman et al., 2004; Bressel et al., 2007).

The relationship between balance performance and the level of expertise flanks the established association between balance ability and risk of sports injury (Hrysomallis, 2007) and highlights the role of postural balance in a sports context. In our study, balance control differences between high-ranking and low-ranking skiers occurred only in the sport-specific dynamic task (Figure 3). The higher the sport specificity of the dynamic balance task, the more it reflects the ranking level of the athletes. The current results also mirror the demands faced by alpine skiers, where effective balance and motor control are required to cope with course setting, terrain variety, and snow conditions (Gilgien et al., 2018). Therefore, in agreement with present results and previous findings, we can speculate that the better dynamic balance of high-ranking skiers could be a factor that can exert considerable influence on the successful performance of alpine ski racers (Ogrin et al., 2021). Our results contrast with those of Noè and Paillard who reported similar static and sport-specific balance performances in skiers at regional and national levels (Noé and Paillard, 2005), and support previous research on the use of sport-specific dynamic balance tests to ensure the proper selectivity among athletes (Asseman et al., 2008). In this regard, the use of an unspecific dynamic balance test (i.e., the star excursion balance test) could explain the non-significant results of Bressel and colleagues who compared collegiate gymnasts and soccer players (Bressel et al., 2007), although some sensorimotor challenges be similar across the disciplines studied.

The better balance performance (i.e., FB, FiB, and GB) in the SST compared to the UST condition (Table 2) is in line with the results of Noè and colleagues (2009), that found higher stability when skiers wore ski boots. We can speculate that wearing ski boots for several hours on the slopes induces young elite skiers to adopt specific postural strategies (i.e., hip and/or knee strategies) that overcome the constraints at the ankle joint. Moreover, the better performance in competitions of the high-ranking skiers could be one of the factors contributing to better performance over the unstable boards, enabling them to enhance or more effectively use their skiing technique in challenging on-snow conditions. We can speculate that high-ranking skiers could have initiated more effective motor control, coordination, and balance strategies in the sport-specific dynamic tasks because of their superior expertise on snow (Tchórzewski et al., 2013).

Finally, we deepened the postural control strategies of the dominant and non-dominant limbs in the SST because, in skiing, each foot is responsible for separately receiving sensory stimuli (Tchórzewski et al., 2013). Our findings indicated that the dominant and non-dominant limbs equally contributed to the management of the SST (Table 4; Figure 4) independently from the skiers’ ranking level. However, the high-ranking group expressed the best balance performance (Table 2) despite the magnitude of ground reaction forces while turning on-snow is different for the inner and the outer leg (Ogrin et al., 2021). Again, we can speculate that high-ranking skiers adopted more effective control strategies to overcome the constraints at the ankle joint.

The present study has some potential limitations that should be considered. Although the group included male and female skiers, it was impossible to perform statistical analyses on sex differences because of the relatively small number of female skiers. In addition, skiers reported a comparable levels of training during the interview but we did not assess the overall amount of physical activity using a validated questionnaire. In addition, balance tests were conducted in a laboratory environment where the room temperature was far from comparable to that on the slopes. Indeed, the warm temperature could have affected the stiffness of the ski boots (Petrone et al., 2014). Moreover, we studied a specific group of young skiers in a growth phase; thus, our results do not necessarily apply to elite adult skiers or athletes of other disciplines.

## Conclusion

Balance ability has been associated with competition level for many sports. However, balance is often assessed using unspecific tests that do not take into consideration sport-specific postures assumed during training and competition. In the present study, skiers demonstrated a better dynamic balance performance in the sport-specific rather than unspecific tests. Moreover, high-ranking skiers showed better balance control and performance than low-ranking skiers only when the task was sport-specific. Therefore, we suggest that balance ability should be tested under sport-specific conditions to better discriminate the skiers’ ability, favoring dynamic over static tests. Future research could provide valuable insight into how the introduction of sport-specific balance tests could help to determine the abilities of young skiers and monitor their improvements during the competitive season.

## Data Availability

The raw data supporting the conclusion of this article will be made available by the authors, without undue reservation.

## Abbreviations

CoP, center of pressure; FB, full balance; FiB, fine balance; GB, gross balance; ST, static test; SST, sport-specific test; UST, unspecific test.
